# Supplementary material for: The assessment of physiotherapy practice is a robust measure of entry-level physiotherapy standards: Reliability and validity evidence from a large, representative sample
Source: PLoS One. 2025 Apr 18;20(4):e0321397. doi: 10.1371/journal.pone.0321397 (PMC12007712; doi:10.1371/journal.pone.0321397)
Supplement: S1 File — (PDF) [file pone.0321397.s002.pdf]

Assessment of Physiotherapy Practice

End of Unit
Summative Assessment

Student name:... **Facility/Experience:**.. **Date**:................

0 = Infrequently/rarely demonstrates performance indicators
1 = Demonstrates few performance indicators to an adequate standard
2 = Demonstrates most performance indicators to an adequate standard
3 = Demonstrates most performance indicators to a good standard
4 = Demonstrates most performance indicators to an excellent standard
not assessed = item was not assessed
***Note.* a rating of 0 or 1 indicates that minimum acceptable competency has not been achieved**

**Professional Behaviour**		**Circle one number only**					
1.	Demonstrates an understanding of client rights and consent	0	1	2	3	4	not assessed
2.	Demonstrates commitment to learning	0	1	2	3	4	not assessed
3.	Demonstrates ethical, legal & culturally responsive practice	0	1	2	3	4	not assessed
4.	Demonstrates collaborative practice	0	1	2	3	4	not assessed
**Communication**							
5.	Communicates effectively and appropriately - Verbal/non-verbal	0	1	2	3	4	not assessed
6.	Demonstrates clear and accurate documentation	0	1	2	3	4	not assessed
**Assessment**							
7.	Conducts an appropriate client-centred interview	0	1	2	3	4	not assessed
8.	Selects and measures relevant health indicators and outcomes	0	1	2	3	4	not assessed
9.	Performs appropriate physical assessment procedures	0	1	2	3	4	not assessed
**Analysis & Planning**							
10.	Appropriately interprets assessment findings	0	1	2	3	4	not assessed
11.	Identifies and prioritises client's problems	0	1	2	3	4	not assessed
12.	Sets realistic short and long term client-centred goals	0	1	2	3	4	not assessed
13.	Selects appropriate intervention in collaboration with the client	0	1	2	3	4	not assessed
**Intervention**							
14.	Performs interventions appropriately	0	1	2	3	4	not assessed
15.	Is an effective educator	0	1	2	3	4	not assessed
16.	Monitors the effect of intervention	0	1	2	3	4	not assessed
17.	Progresses intervention appropriately	0	1	2	3	4	not assessed
18.	Undertakes discharge planning	0	1	2	3	4	not assessed
**Evidence-based Practice**							
19.	Applies evidence based practice in client-centred care	0	1	2	3	4	not assessed
**Risk Management**							
20.	Identifies adverse events/near misses and minimises risk associated with assessment and interventions	0	1	2	3	4	not assessed

In your opinion as a clinical educator, the overall performance of this student in the clinical unit was:

Not adequate Adequate Good Excellent

Examples of Performance Indicators

Professional Behaviour

1. Demonstrates an understanding of client rights and consent

- obtains & records informed consent according to protocol
- recognises clients' health-care rights
- prioritises clients' rights, needs & interests
- allows sufficient time to discuss the risks & benefits of the proposed treatment with clients & carers
- refers clients to a more senior staff member for consent when appropriate
- advises supervisor or other appropriate person if a client might be at risk
- respects clients' privacy & dignity
- complies with confidentiality & privacy requirements for client's health & personal information
- applies ethical principles to the collection, maintenance, use & dissemination of data & information

2. Demonstrates commitment to learning

- responds in a positive manner to questions, suggestions &/or constructive feedback
- reviews & prepares appropriate material before & during placement
- develops & implements a plan of action in response to feedback
- seeks information/assistance as required
- demonstrates self-evaluation, reflects on progress & implements appropriate changes based on reflection
- takes responsibility for learning & seeks opportunities to meet learning needs
- uses clinic time responsibly

3. Demonstrates ethical, legal & culturally responsive practice

- follows policies & procedures of the facility
- advises appropriate staff of circumstances that may affect adequate work performance
- observes infection control, & workplace health & safety policies
- arrives fit to work
- arrives punctually & leaves at agreed time
- calls appropriate personnel to report intended absence
- wears an identification badge & identifies self
- recognises inappropriate or unethical health practice
- observes dress code
- completes projects/tasks within designated time frame
- maintains appropriate professional boundaries with clients & carers
- advocates for clients & their rights (where appropriate)
- demonstrates appropriate self-care strategies (e.g. management of stress, mental & physical health issues)
- acts ethically & applies ethical reasoning in all health care activities
- demonstrates skills in culturally safe & responsive client-centred practice
- acts within bounds of personal competence, recognizing personal & professional strengths & limitations

4. Demonstrates collaborative practice

- demonstrates understanding of team processes
- contributes appropriately in team meetings
- acknowledges expertise & role of other health care professionals & refers/liaises as appropriate to access relevant services
- advocates for the client when dealing with other services
- collaborates with the health care team & client to achieve optimal outcomes
- cooperates with other people who are treating & caring for clients
- guides & motivates support staff (where appropriate)
- works collaboratively & respectfully with support staff

Communication

5. Communicates effectively and appropriately - Verbal/non-verbal

- greets others appropriately
- questions effectively to gain appropriate information
- listens carefully & is sensitive & empathetic to views of client & relevant others
- respects cultural & personal differences of others
- gives appropriate, positive reinforcement
- provides clear instructions
- uses suitable language & avoids jargon
- demonstrates an appropriate range of communication styles (with e.g. clients, carers, administrative & support staff, health professionals, care team)
- recognises barriers to optimal communication
- responds appropriately to non-verbal cues
- integrates communication technology into practice as required
- uses a range of communication strategies to optimize client rapport & understanding (e.g. hearing impairment, non-English speaking, cognitive impairment, consideration of non-verbal communication)
- uses accredited interpreters appropriately
- maintains effective communication with clinical educators
- recognises risk of conflict & takes appropriate action to mitigate &/or resolve
- actively explains to clients & relevant others their role in care, decision-making & preventing adverse events
- actively encourages clients to provide complete information without embarrassment or hesitation
- conducts communication with client in a manner & environment that demonstrates consideration of confidentiality, privacy & client's sensitivities
- negotiates appropriately with other health professionals

6. Demonstrates clear and accurate documentation

- writes legibly
- completes relevant documentation to the required standard (e.g. client record , statistical information, referral letters)
- maintains records compliant with legislative medico-legal requirements
- complies with organisational protocols & legislation for communication
- adapts written material for a range of audiences (e.g. provides translated material for non-English speaking people, considers reading ability, age of client)

Assessment

7. Conducts an appropriate client interview

- positions person safely & comfortably for interview
- structures a systematic, purposeful interview seeking qualitative & quantitative details
- provides a culturally safe environment for the client
- asks relevant & comprehensive questions
- politely controls the interview to obtain relevant information
- responds appropriately to important client cues
- identifies client's goals & expectations
- conducts appropriate assessment with consideration of the social, personal, environmental & biopsychosocial factors that influence function, health & disability.
- seeks appropriate supplementary information, accessing other information, records, test results as appropriate & with client's consent
- generates diagnostic hypotheses, identifying priorities & urgency of further assessment & intervention
- completes assessment in acceptable time

8. Selects and measures relevant health indicators and outcomes

- selects appropriate variable/s to be measured at <u>baseline</u> from WHO ICF domains of impairment, activity limitation & participation restriction.
- identifies & justifies variables to be measured to <u>monitor</u> treatment response & outcome.
- selects appropriate <u>tests/outcome measures</u> for each variable for the purpose of diagnosis, monitoring & outcome evaluation.
- links outcome variables with treatment goals
- communicates the treatment evaluation process & outcomes to the client & relevant others
- identifies, documents & acts on factors that may compromise treatment outcomes

9. Performs appropriate physical assessment procedures

- considers client comfort & safety
- respects client's need for privacy & modesty (e.g. provides draping or gown)
- structures systematic, safe & goal oriented assessment processes accommodating limitations imposed by client's health status
- plans assessment structure & reasoning process using information from client history & supportive information

Examples of Performance Indicators

- demonstrates sensitive & appropriate handling during the assessment process
- applies tests & measurements safely, accurately & consistently
- sensibly modifies assessment in response to client profile, feedback & relevant findings
- performs appropriate tests to refine diagnosis
- assesses/appraises work, home or other relevant environments as required
- completes assessment in acceptable time

Analysis & Planning

10. Appropriately interprets assessment findings

- describes the implications of test results
- describes the presentation & expected course of common clinical conditions
- relates signs & symptoms to pathology
- relates signs, symptoms & pathology to environmental tasks & demands
- interprets findings at each stage of assessment to progressively negate or reinforce hypothesis/es
- makes justifiable decisions regarding diagnoses based on knowledge & clinical reasoning
- prioritises important assessment findings
- compares observed findings to expected findings

11. Identifies and prioritises client's problems

- generates a list of problems from the assessment
- justifies prioritisation of problem list based on knowledge & clinical reasoning
- collaborates with client to prioritise problems
- considers client's values, priorities & needs

12. Sets realistic short and long term goals with the client

- negotiates realistic short term treatment goals in partnership with client
- negotiates realistic long term treatment goals in partnership with client
- formulates goals that are specific, measurable, achievable & relevant, with specified timeframe
- considers physical, emotional & financial costs, & relates them to likely gains of intervention

13. Selects appropriate intervention in collaboration with the client

- engages with client to explain assessment findings, discuss intervention strategies & develop an acceptable plan
- identifies & justifies options for interventions based on client needs, clinical guidelines, best evidence & available resources
- considers whether physiotherapy is indicated
- demonstrates a suitable range of skills & approaches to intervention
- describes acceptable rationale (e.g. likely effectiveness) for treatment choices
- balances needs of clients & relevant others with the need for efficient & effective intervention
- demonstrates understanding of contraindications & precautions in selection of intervention strategies
- advises client about the effects of treatment or no treatment

Intervention

14. Performs interventions appropriately

- considers the scheduling of treatment in relation to other procedures e.g. medication for pain, wound care.
- demonstrates appropriate client handling skills in performance of interventions
- performs techniques at appropriate standard
- minimizes risk of adverse events to client & self in performance of intervention (including observance of infection control procedures & manual handling standards)
- prepares environment for client including necessary equipment for treatment
- identifies when group activity might be an appropriate intervention
- demonstrates skill in case management
- recognises when to enlist assistance of others to complete workload
- completes intervention in acceptable time
- refers client to other professional/s when physiotherapy intervention is not appropriate, or requires a multi-disciplinary approach

15. Is an effective educator

- demonstrates skill in client education & health promotion e.g. modifies approach to suit client age group &/or cultural needs
- applies adult learning principles in education of clients & relevant others
- educates assistants & relevant others to implement safe & effective therapy
- participates in leading educational activities for peers/staff (where appropriate)
- demonstrates skills in conducting group sessions
- develops a realistic self-management program for prevention & management in collaboration with the client
- provides information using a range of strategies that demonstrate consideration of client needs
- confirms client's/relevant others' understanding of given information
- uses appropriate strategies to motivate the client & relevant others to participate & to take responsibility for achieving defined goals
- discusses expectations of physiotherapy intervention & its outcomes
- provides feedback to client regarding health status
- educates the client in self evaluation
- encourages & acknowledges achievement of short & long term goals

16. Monitors the effects of intervention

- incorporates relevant evaluation procedures/outcome measures in the physiotherapy plan
- monitors client response to the intervention
- makes modifications to intervention based on therapist evaluation & client feedback
- records & communicates outcomes where appropriate

17. Progresses intervention appropriately

- demonstrates or describes safe & sensible treatment progressions
- makes decisions regarding modifications, continuation or cessation of intervention in consultation with the client, based on best available evidence
- discontinues treatment in the absence of measurable benefit

18. Undertakes discharge planning

- begins discharge planning in collaboration with the health care team at the time of the initial episode of care
- discusses discharge planning with the client
- describes strategies that may be useful for maintaining or improving health status following discharge
- arranges appropriate follow-up health care to meet short & long term goals
- addresses client & carer needs for ongoing care through the coordination of appropriate services

Evidence Based Practice

19. Applies evidence based practice in client-centred care

- considers the research evidence, client preferences, clinical expertise & available resources in making treatment decisions & advising clients
- practises in accordance with relevant clinical practice guidelines
- locates & applies relevant current evidence e.g. clinical practice guidelines & systematic reviews
- assists clients & carers to identify reliable & accurate health information
- shares new evidence with colleagues
- participates in & applies quality improvement procedures when possible

Risk Management

20. Identifies adverse events and nearmisses and minimises risk associated with assessment and interventions

- monitors client safety during assessment & treatment
- complies with workplace guidelines on manual handling
- complies with organizational health & safety requirements
- describes relevant contraindications & precautions associated with assessment & treatment
- recognises & reports adverse events & near misses to appropriate members of the team
- implements appropriate measures in case of emergency
- reports inappropriate or unsafe behaviour of a co-worker or situations that are unsafe
- prior to client contact, reports any personal issues (physical/mental) that may impact on client care
