# Supplementary material for: The assessment of physiotherapy practice is a robust measure of entry-level physiotherapy standards: Reliability and validity evidence from a large, representative sample
Source: PLoS One. 2025 Apr 18;20(4):e0321397. doi: 10.1371/journal.pone.0321397 (PMC12007712; doi:10.1371/journal.pone.0321397)
Supplement: Attachment — Submitted filename: PONE-D-24-38498_response to reviewers.docx [file pone.0321397.s005.docx]

**ACADEMIC EDITOR**

|  | **Reviewer’s Comments** | **Our Response and Location in Revised Manuscript** |
| --- | --- | --- |
| **1** | Please ensure that your manuscript meets PLOS ONE's style requirements, including those for file naming. The PLOS ONE style templates can be found [here](https://journals.plos.org/plosone/s/file?id=wjVg/PLOSOne_formatting_sample_main_body.pdf) and [here](https://journals.plos.org/plosone/s/file?id=ba62/PLOSOne_formatting_sample_title_authors_affiliations.pdf). | We’ve audited the manuscript and supporting documentation to ensure they adhere to the journal guidelines. As two examples, we’ve replaced the word ‘figure’ with ‘fig’ within the manuscript itself, and updated the title page of the manuscript.  We have also uploaded Fig 1 as a .tif file. |
| **2** | Please include a complete copy of PLOS’ questionnaire on inclusivity in global research in your revised manuscript. Our policy for research in this area aims to improve transparency in the reporting of research performed outside of researchers’ own country or community. The policy applies to researchers who have travelled to a different country to conduct research, research with Indigenous populations or their lands, and research on cultural artefacts. The questionnaire can also be requested at the journal’s discretion for any other submissions, even if these conditions are not met.  Please find more information on the policy and a link to download a blank copy of the questionnaire here: [https://journals.plos.org/plosone/s/best-practices-in-research-reporting](https://aus01.safelinks.protection.outlook.com/?url=https%3A%2F%2Fjournals.plos.org%2Fplosone%2Fs%2Fbest-practices-in-research-reporting&data=05%7C02%7Cd.gucciardi%40curtin.edu.au%7Ca9d1e674c615480c753908dcf7ee5497%7C5a740cd757684d09ae13f706b09fa22c%7C0%7C0%7C638657850201577302%7CUnknown%7CTWFpbGZsb3d8eyJWIjoiMC4wLjAwMDAiLCJQIjoiV2luMzIiLCJBTiI6Ik1haWwiLCJXVCI6Mn0%3D%7C0%7C%7C%7C&sdata=u%2Bh%2F88JCRS4p3DwdP7v3jP%2FykWLpgHGqfX4rK%2FRcJfQ%3D&reserved=0). Please upload a completed version of your questionnaire as Supporting Information when you resubmit your manuscript. | We’ve added the following text to the Methods section and attached the requested survey:  ***“Inclusivity in global research*** *Additional information regarding the ethical, cultural, and scientific considerations specific to inclusivity in global research is included in the Supporting Information (S1 Checklist)”* |
| **3** | We note that you have indicated that there are restrictions to data sharing for this study. For studies involving human research participant data or other sensitive data, we encourage authors to share de-identified or anonymized data. However, when data cannot be publicly shared for ethical reasons, we allow authors to make their data sets available upon request.  Before we proceed with your manuscript, please address the following prompts:    a) If there are ethical or legal restrictions on sharing a de-identified data set, please explain them in detail (e.g., data contain potentially identifying or sensitive patient information, data are owned by a third-party organization, etc.) and who has imposed them (e.g., a Research Ethics Committee or Institutional Review Board, etc.). Please also provide contact information for a data access committee, ethics committee, or other institutional body to which data requests may be sent.    b) If there are no restrictions, please upload the minimal anonymized data set necessary to replicate your study findings to a stable, public repository and provide us with the relevant URLs, DOIs, or accession numbers. Please see [http://www.bmj.com/content/340/bmj.c181.long](https://aus01.safelinks.protection.outlook.com/?url=http%3A%2F%2Fwww.bmj.com%2Fcontent%2F340%2Fbmj.c181.long&data=05%7C02%7Cd.gucciardi%40curtin.edu.au%7Ca9d1e674c615480c753908dcf7ee5497%7C5a740cd757684d09ae13f706b09fa22c%7C0%7C0%7C638657850201606404%7CUnknown%7CTWFpbGZsb3d8eyJWIjoiMC4wLjAwMDAiLCJQIjoiV2luMzIiLCJBTiI6Ik1haWwiLCJXVCI6Mn0%3D%7C0%7C%7C%7C&sdata=WvWHkWw1MavY2KmzjXZybmMktENoO6WxmXXwwHck528%3D&reserved=0) for guidelines on how to de-identify and prepare clinical data for publication. For a list of recommended repositories, please see [https://journals.plos.org/plosone/s/recommended-repositories](https://aus01.safelinks.protection.outlook.com/?url=https%3A%2F%2Fjournals.plos.org%2Fplosone%2Fs%2Frecommended-repositories&data=05%7C02%7Cd.gucciardi%40curtin.edu.au%7Ca9d1e674c615480c753908dcf7ee5497%7C5a740cd757684d09ae13f706b09fa22c%7C0%7C0%7C638657850201621301%7CUnknown%7CTWFpbGZsb3d8eyJWIjoiMC4wLjAwMDAiLCJQIjoiV2luMzIiLCJBTiI6Ik1haWwiLCJXVCI6Mn0%3D%7C0%7C%7C%7C&sdata=csCPyvgNV6F6aGqvrSzamZR7moqnIqjgEex%2FrOW9fRA%3D&reserved=0). You also have the option of uploading the data as Supporting Information files, but we would recommend depositing data directly to a data repository if possible.    Please update your Data Availability statement in the submission form accordingly. | Curtin University's Human Research Ethics Committee approved the study (HRE2021-0333) and provided approval for consent waiver. All participating universities gained reciprocal ethics and other governance approvals as required. Given the sensitive nature of our data and that students never provided consent for their student academic records to be made public, our ethics committee required us to keep raw data confidential and therefore we are unable to share this data publicly. Interested readers can contact Curtin University’s Human Research Ethics Committee ([hrec@curtin.edu.au](mailto:hrec@curtin.edu.au)) for requests to access the raw data.  We have added the following to the Data Availability Statement, with red text denoting new information within our revised submission:  *The participants of this study did not provide written consent for their data to be shared publicly. Due to the sensitive nature of the data, namely student academic records, supporting data is unavailable. Data are available on request from the Curtin University Human Research Ethics Committee (*[*hrec@curtin.edu.au*](mailto:hrec@curtin.edu.au)*) for researchers who meet the criteria to gain access to confidential data*.  As noted in our original submission, we’ve made public all input and output files for the statistical models reported in the manuscript (<https://bit.ly/PCERC-app>). |
| **4** | One of the noted authors is a group [Physiotherapy Clinical Education Research Collaborative (PCERC)]. In addition to naming the author group, please list the individual authors and affiliations within this group in the acknowledgments section of your manuscript. Please also indicate clearly a lead author for this group along with a contact email address. | We provided names and affiliations of the Physiotherapy Clinical Education Research Collaborative (PCERC) on the title page of our original submission. As corresponding author of this paper, Alan Reubenson is the lead author for the PCERC. |
| **5** | Your ethics statement should only appear in the Methods section of your manuscript. If your ethics statement is written in any section besides the Methods, please move it to the Methods section and delete it from any other section. Please ensure that your ethics statement is included in your manuscript, as the ethics statement entered into the online submission form will not be published alongside your manuscript. | An ‘Ethics statement’ sub-heading and text has been added to the Methods section, after the ‘Design’ sub-heading. |
| **6** | Please include captions for your Supporting Information files at the end of your manuscript, and update any in-text citations to match accordingly. Please see our Supporting Information guidelines for more information: [http://journals.plos.org/plosone/s/supporting-information](https://aus01.safelinks.protection.outlook.com/?url=http%3A%2F%2Fjournals.plos.org%2Fplosone%2Fs%2Fsupporting-information&data=05%7C02%7Cd.gucciardi%40curtin.edu.au%7Ca9d1e674c615480c753908dcf7ee5497%7C5a740cd757684d09ae13f706b09fa22c%7C0%7C0%7C638657850201636078%7CUnknown%7CTWFpbGZsb3d8eyJWIjoiMC4wLjAwMDAiLCJQIjoiV2luMzIiLCJBTiI6Ik1haWwiLCJXVCI6Mn0%3D%7C0%7C%7C%7C&sdata=PX8e21MISNGs8IumHaOL5P8ZNyW4eO7tTVMQGIbigSk%3D&reserved=0). | We’ve updated our reporting of the Supporting Information materials in the manuscript according to the journal guidelines (e.g., see Results section) and added the requested section at the end of the manuscript. |

**REVIEWER 1**

|  | **Reviewer’s Comments** | **Our Response and Location in Revised Manuscript** |
| --- | --- | --- |
|  | Great paper of significant value to the physiotherapy profession. I think to make this more accessible to student supervisors and physiotherapy academics the language needs to be simplified, or complex terms given context. The other key factors that I believe needs to be explained further is why/how modelling the assessment outcomes improves generalisability and how this can be applied by supervisors and academic staff in assessment. I think the statistical approach taken in rigorous it just needs to justified more clearly. For example, with research question one it is not quite clear from the paper why we need to distil the APP down to one of two factors. This question also mentions clinical performance, but the other element explored is professionalism. | We appreciate the time and effort you allocated to reviewing our manuscript, and your constructive feedback regarding how we might strengthen the final product. For context, we consider the peer review process an academic discussion, so provide substantively and/or empirically justified responses where we disagree with your recommendations. For all other cases, we’ve included snippets of text in this response document to show how we’ve actioned your feedback.  We respond here to four of your general points:   1. **The language needs to be simplified or context provided for complex terms.** Thanks for this recommendation. We’ve addressed this general comment in our revised submission in various places. Two broad approaches are worth mentioning explicitly. First, we’ve limited our use of the term ‘factor’ to instances where we discuss the statistical analyses or outputs, rather than conceptual or substantive descriptions of determinants, contextual characteristics, and so on. As one example, we replaced the term ‘factor’ with ‘considerations’ in the opening paragraph of the introduction:   “*The Assessment of Physiotherapy Practice (APP) was developed as a standardised instrument, within Australia and New Zealand (ANZ), for the assessment and evaluation of physiotherapy student performance against the same entry-level standards across diverse contextual factors considerations* [3-5].”  Second, we have revised the wording of the research questions to refer to ‘entry-level physiotherapy student performance’ instead of using ‘clinical performance’ to avoid confusion with the two elements of ‘professionalism’ and ‘clinical’ within the APP.  *1. Do one or two factors best represent entry-level physiotherapy student performance scores obtained via the APP and what is the nature of their characterisation?*  *2. To what extent are the same number of factors and their interpretation, and item scaling captured equally over time and across contexts for assessments of entry-level physiotherapy student performance via the APP?*  Where appropriate, within the body of the manuscript, we have also used similar terminology to link back to this e.g.,  *Assessment of student physiotherapy practice performance is a complex endeavour that has evolved substantially over the past half century [24].*   2. **Why and how does the modelling approach improve generalisation**? As noted in the introduction section, the acquisition and analysis of a representative sample of clinical performance assessments is the first core element to the generalisation claims of this work. We’ve expanded upon elements of the introduction to clarify the links between a representative sample and generalisation, and forecast the centrality of our statistical model.   *Owing to its success in related areas of the human sciences [30], we took a ‘Big Team’ science approach to maximise inferences regarding the generalisation of evidence, whereby a large group of collaborators worked together to address a common goal [31]. In 2020, approximately 40 university staff and researchers across 23 ANZ universities established the Physiotherapy Clinical Education Research Collaborative (PCERC). This collaboration created a large and broad representative sample of entry-level physiotherapy placement activity across ANZ. In so doing, our representative sample of physiotherapy students undertaking real-world placements maximises generalisation because the findings are applicable to the broader population and breadth of placement environments, and reduces bias, increases external validity, facilitates meaningful comparisons, supports statistical inference, and enhances the overall credibility of the research. We complement this sampling approach to representativeness with a statistical framework that permits direct tests of the extent to which our findings generalise across people, time, and context*.  Statistically, measurement invariance analyses provide the analytical framework upon which to examine the extent to which test scores generalise across people, time, and context. We’ve expanded upon the information provided in the analysis sub-section of the methods section to clarify the link with generalisation.  *Meaningful group comparisons using aggregate test scores rest on the assumption that latent variables are captured via the same origin and scale and therefore share the same operational definition and meaning across (sub)populations [38, 39]. Achieving measurement invariance is crucial for comparing scores between groups or over time, as it ensures that observed differences reflect true variations in the construct rather than inconsistencies in measurement. The subpopulations of interest within our sample include repeated assessments and three elements that characterise contextual features of the professional practice landscape within ANZ, namely placement type (cardiorespiratory, musculoskeletal, neurological, other, or some combination of them) and setting (hospital-inpatient, hospital-outpatient, hospital-unknown, or other), and the year in which placements occurred (final year only or penultimate and final years). Measurement invariance evidence is essential for inferences regarding the generalisation of concepts and assessment properties. Evidence that supports measurement invariance across time and context provides confidence that assessment instruments function roughly equivalently irrespective of the circumstances in which they are applied*.   3. **How can the modelling approach be applied by supervisors and staff**? We’re unsure the statistical modelling approach itself is necessary for clinical supervisors who provide these performance assessments of students. In essence, their task is to assess students according to the behavioural indicators captured by each of the 20 APP items. The statistical modelling approach is 100% relevant for academic staff at universities involved in the integration of APP data for making inferences regarding student performance for summative scores in clinical units. However, it’s unlikely that most physiotherapy academics will possess the expertise required to execute psychometric analyses in the absence of additional training and/or someone on staff who already holds such expertise. An app powered by the Shiny package for conducting psychometric analyses on new data and integrating that evidence interactively with existing data is something our collaboration is discussing and debating. See this paper ([Allbritton et al., 2024](https://journalofcognition.org/articles/10.5334/joc.389)) and the associated app ([here](https://dallbrit.shinyapps.io/Breathing_Life_into_MetaAnalysis/)) for an example within the context of dynamic, living meta-analyses. In contrast, the findings of our research can inform practice and processes on the ground by supervisors and staff. We’ve flagged a couple of possibilities in our discussion, as noted below.   *We showed that interpretation of entry-level physiotherapy performance is best operationalised via the APP as a 2-factor concept with domains characterised by professional (items 1-4) and clinical (items 5-20) indicators. The 2-factor representation aligns with contemporary practice, whereby healthcare graduates should be professional, ethically, and legally responsible as well as technically-skilled. Delivering physiotherapy care via assessment, analysis, and planning together with high-quality professional behaviours such as patient rights and consent is essential for safe and effective practice, as per contemporary standards for entry-level performance and registration requirements [15, 17]. The 2-factor representation also enhances the utility of the APP for educators and supervisors by highlighting practice areas where students may need focussed support or development. In summary, to meet entry-level standards physiotherapy students should be assessed as competent across both professional and clinical dimensions of physiotherapy practice*.   4. **Why do we need to distil the APP down to one or two factors**? We noted in the introduction, “Collectively, the available evidence suggests entry-level competency as assessed by the APP can be considered a global or two-dimensional concept.” Thus, our prioritisation on 1 or 2-factor solutions is guided primarily by evidence (including previous publications e.g., see references [6-9]). The labelling of these dimensions as professional or clinical is guided primarily by inferences regarding the thematic content of items that load on those latent factors. We’ve added the following statement to the introduction section to clarify this point.   *The APP was designed to provide a unidimensional assessment of entry-level competency, whereby 20 items are aggregated into a single factor or total score representing overall minimal clinical competence to practice [3, 4]. However, alternative interpretations suggest that overall physiotherapy entry-level competence could be better represented by two dimensions: professional behaviours (items 1-4) and clinical skills (items 5-20) [7]. The descriptive labelling of these dimensions as ‘professional’ and ‘clinical’ stems from thematic analysis of item content and indicators associated with each factor. Given this evidence, there is an ongoing question of whether entry-level competency is best captured as a global, single-factor or two-dimensional construct comprised of professional and clinical factors*. |
|  | Abstract |  |
| **1** | Abstract is well structured. I know there it is difficult with a restricted word count but the reason for separating out the 2 dimensions could be explained a little more. It is not clear if this was the intention during the original design of the APP or done for the purpose of this study. | We’ve clarified in the abstract that the original reliability and validity work supported a unidimensional or single latent factor structure for the APP.  *Initial APP reliability and validity evidence supported a unidimensional or single latent factor as the best representation of entry-level physiotherapy practice performance. However, there remains inconsistency in how the APP is interpreted and operationalised across Australian and New Zealand universities offering entry-level physiotherapy programs*.  It is clear from the description of our results statement in the abstract that our solution is primarily statistically informed – we’ve bolded keywords below for emphasis.  ***Structural representation*** *of APP scores were examined* ***via confirmatory factor analysis and penalised structural equation models****.* ***Factor analyses*** *indicated a 2-factor representation, with four items (1-4) for the professional dimension and 16 items (5-20) for the clinical dimension, is the best approximation of entry-level physiotherapy performance.* ***Measurement invariance analyses*** *supported the robustness of this 2-factor representation over time and across diverse practice areas in both penultimate and final years of study*. |
|  | Introduction |  |
| **2** | Page 7, 2nd sentence is long and compound. Could this be broken up for clarity as these are important foundational point. | We have broken up this statement into two segments, as outlined below:  *In these settings, students can test and apply previously acquired skills and knowledge. Under the supervision of registered physiotherapists, students provide care to a diverse range of patients and clients*. |
| **3** | The next sentence beginning ‘the authentic workplace’ needs context, it is no clear what when consider holistically refers to. | We’ve updated this statement to provide the necessary context for key terms in this statement.  *The authentic workplace environment can be a powerful and transformative learning space for students when all aspects such as practical experience, mentorship, and collaboration are considered holistically and alongside application of academic knowledge with real-world application*. |
| **4** | The next sentence also needs contextualisation, the practice development crucible needs explanation. It alludes to four key intersecting influences but does not go on to explain what these are. I am assuming they are contained in the next sentence, but this is not clear. | We’ve updated this statement to provide the necessary context for key terms in this statement.  *The multidimensional nature of workplace learning can be considered using a practice development crucible metaphor, which illustrates how various considerations interact to shape student learning experiences [20]. This metaphor encompasses four key intersecting influences: (1) workplace influences, which include physical resources and workplace culture; (2) engagement in professional practices, highlighting the importance of student interactions with patients and staff; (3) clinical supervisors’ intentions and actions, reflecting how supervisors’ approaches influence student learning; and (4) students’ dispositions and experiences, which encompass students’ confidence and motivation. Understanding these influences is essential for maximising student learning in clinical placements. Students’ dispositions and prior experiences including their knowledge, skills, and self-reflection, as well as characteristics such as confidence and engagement within the multi-disciplinary team play a crucial role in their learning. Nevertheless, these independent influences rarely occur in isolation and should be understood within the broader context of workplace elements, such as the culture and practices of the workplace, as well as the actions and intentions of clinical supervisors, like the quality of feedback they provide and their motivation to support student learning [23]. Therefore, evaluating student performance in these environments requires a comprehensive approach that considers all these interconnected elements*. |
| **5** | My opinion, but I find the use of frequent use of bracketed examples (eg…) distracting to read. If these were removed and the example incorporated into the text, this would improve flow. | Thanks for this personal perspective on our writing approach and style. We’ve audited the manuscript to remove most but not all instances of bracketed examples. In so doing, we rely on bracketed examples sparingly, reserving them for particularly complex points where clarity is essential. See below for one example of how we have actioned this recommendation:  *Students’ dispositions and prior experiences (e.g., knowledge, skills, self-reflection), and professional practice engagement (e.g., confidence and interactions within the multi-disciplinary team) are critical*.  *Students’ dispositions and prior experiences including their knowledge, skills, and self-reflection, as well as characteristics such as confidence and engagement within the multi-disciplinary team play a crucial role in their learning*. |
| **6** | Pg 7: the sentence beginning “Nevertheless, these independent…” needs further simplification of terms. Concepts such as multidimensional and holistic have not been established in the context of this paper. | Thanks for this point. We’ve amended this text so that is excludes the use of multidimensional and holistic, and links with the key components of the crucible metaphor outlined in the previous statement of this paragraph.  *Nevertheless, these independent influences rarely occur in isolation and should be understood within the broader context of workplace elements, such as the culture and practices of the workplace, as well as the actions and intentions of clinical supervisors, like the quality of feedback they provide and their motivation to support student learning [23]. Therefore, evaluating student performance in these environments requires a comprehensive approach that considers all these interconnected elements*. |
| **7** | Last sentence of page 7 can be simplified – I don’t think this needs to be positioned within a sociocultural framework, it just is what it is – or explain what is meant in this context by a socio-cultural framework. | Thanks for this point. We’ve reshaped this section of the manuscript to clarify our position on socio-cultural considerations.  *Assessment of student physiotherapy practice performance is a complex endeavour that has evolved substantially over the past half century [24]. In the health sciences, work-based assessments are now the primary method for evaluating performance in real-world practice settings through direct observation [25, 26]. In these contexts, assessment processes are influenced by the social and cultural contexts in which they occur, including interactions between students, supervisors, and the broader healthcare environment, all of which shape feedback and learning experiences. Work-based assessment within this framework relies on both summative and formative feedback processes to determine the achievement of professional standards* [24, 26]. |
| **8** | Second sentence of page 8 needs referencing | We presume you refer to the following statement:  *In the physiotherapy discipline, evaluation of clinical performance commonly utilises a longitudinal assessment process, whereby student performance is observed repeatedly over a period of 4-6 weeks, by one or more clinical supervisors*.  If so, we disagree with your recommendation here. Our author team includes representation from most of the ANZ universities offering entry-level physiotherapy programs. In particularly, our team includes numerous Directors of Clinical Education, who meet regularly to discuss the clinical education landscape in ANZ, and drive the policies and processes regarding these learning contexts. Thus, we believe our team is well justified to make this statement, particularly as we acknowledge there are potential exceptions to the rule via the term ‘commonly’ in this statement. |
| **9** | Next sentence beginning with “sampling process” I would disagree it is a sampling process, it is just an assessment process. I would also disagree it considers “the evolving and dynamic nature of complex workplaces” – I think there needs to be careful consideration of simplifying the language in this intro as it can imply meaning that is not intended or needs to be contextualised. | As noted above to your general comments, we’ve audited the manuscript to simply terminology or provide additional context where required. We’ve also amended the terminology for the specific statement in your feedback as per your recommendation.  *This assessment process allows for variation in performance over time and under different circumstances and contexts*.  Regarding the second point, it’s unclear from your feedback why you disagree with our statement that assessment processes consider the evolving and dynamic nature of complex workplaces. Nevertheless, we’ve amended this text in the revised submission to clarify the intent of this statement in relation to assessment processes.  *It also acknowledges that workplaces and healthcare often vary, particularly regarding considerations like culture, policies, and processes as well as the relational interactions with supervisors and patients*. |
| **10** | I think the main paragraph on page 9 is a bit superfluous, The first part about generalisations can be simplified into a research aim and the justified. So can the part about the research collaboration, it is currently somewhat overstated. It is great that the research was a collaboration with leaders in this area, but this is evident from the list of authors and in a study of this nature has little bearing on the findings. | We appreciate your feedback on this concluding paragraph on the introduction section. Our intent with this paragraph is to pull together information that was covered elsewhere in the introduction with broader scientific concepts and processes. Your recommendation to convert the information on generalisation to a research aim is an alternative approach to the one we adopted in the original submission. However, we prefer our existing approach because the essence of generalisation is captured in our questions, particularly research question 2 – “captured equally over time and across contexts”. The text we provide in this section situates generalisation broadly to provide readers with context key elements that are inherent within our design and test combination.  Regarding your point about overstating the collaboration element, we’re unsure exactly where and how we convey this perception. Yes, the long list of co-authors alludes to a large-scale collaborative effort, yet it excludes context regarding the ‘why’ and ‘how’. We believe this information is important to report as one example of how big team science can be operationalised, particularly as this output represents one of the first exemplars of this approach for the physiotherapy profession outside of a clinical trial or intervention context (e.g., multisite trials). We also disagree with your comment that the collaborative approach has little bearing on the findings. Our strategic approach to collaboration enabled us to assemble a “large and broad representative sample of entry-level physiotherapy placement activity across ANZ.” Representative samples are one of several characteristics of high-quality data. Consider the scenario where each individual institution published their own psychometric data on the APP independently; this approach would be incremental, and likely criticised as minimally representative because it reflects data from 1 of 25 sites in the ANZ. Our collaborative approach fast-tracked this knowledge accumulation process by bringing together ~90% of eligible institutions and integrating individual efforts for a representative sample. On the contrary, the collaborative has directly influenced the findings because they are based on a representative sample. |
|  | Methods  The methods section is well written. | We appreciate your positive feedback on the methods. |
| **11** | The outcome measure section does not mention the global rating scale on the APP which is often used as an overall indication of student performance and in some cases represents 20% of the overall student mark. Why was this not included, especially given it may be highly relevant to research question 1. | We’ve never heard of instances where the global rating score contributes 20% of the overall student mark. Our author team represents a large percentage of the ANZ institutions in which the APP is used as the primary assessment for entry-level physiotherapy performance. If the global rating score is being used in this way, it represents the exception rather than rule. Furthermore, attributing 20% of the overall student mark to the global rating scale is incorrect and never recommended as part of the original research and development of the APP.  Individual items refer to specific competencies with associated behaviours or performance indicators, whereas the global rating reflects student performance from an overall or holistic perspective (<https://appeducation.com.au/faq.html>). We prioritised the 20 items rather than the global scale because they directly align with the entry-level competencies and practice thresholds required for registration with the respective Physiotherapy Boards within ANZ. Clinical educators are also advised to complete the global rating scale after they have assessed students on the individual 20 items (<https://www.clinedaus.org.au/files/resources/2012_app_resource_manual_1.pdf>). Thus, it is likely that assessment information regarding the individual items is captured within the global rating. For example, the latent factor correlations between the global rating with professional (r = .71) and clinical (r = .87) dimensions broadly support this expectation. |
|  | Results and Discussion |  |
| **12** | Please see general comments above as these are relevant to the findings and discussion section. | Our interpretation of your feedback above is that we need to audit the results and discussion sections for:   1. Use of parentheses for context within individual sentences 2. Complex terms that require context or elaboration   We’ve audited these section and updated text where appropriate. In the absence of specific guidance, we’ve relied on our author team to flag instances where they believe these points could be addressed. |
| **13** | There appears to be some new information presented in the discussion for example sections of the paragraph that begins on page 17. This should be reviewed. | Our perspective is that there is no new information presented in this section. Item-level factor loadings and model-data fit indices are presented in the results section (Tables 3 and 4). We’ve added the following statement to the revised submission as a briefly inferential summary of those data alongside the signpost in parentheses included in the original submission.  *Most APP items loaded meaningfully onto their respective latent factor (>.40), with higher and more consistent loadings observed for the clinical dimension, and few substantial cross-loadings (e.g., items 2 and 20). The latent factor correlation between the professional and clinical dimensions was moderately strong*.  We simply discuss these findings with additional context for interpretation purposes, relative to our research questions. Perhaps it is these discussion points – such as the continuum of subjective-objective for performance assessments – that you refer to as new information. This contextualisation and elaboration of key findings is entirely the point of a discussion section. |
