# Supplementary material for: The assessment of physiotherapy practice is a robust measure of entry-level physiotherapy standards: Reliability and validity evidence from a large, representative sample
Source: PLoS One. 2025 Apr 18;20(4):e0321397. doi: 10.1371/journal.pone.0321397 (PMC12007712; doi:10.1371/journal.pone.0321397)
Supplement: Attachment — Submitted filename: PONE-D-24-38498_response_to_reviewers_auresp_2.docx [file pone.0321397.s006.docx]

**REVIEWER 2**

|  | **Reviewer’s Comments** | **Our Response and Location in Revised Manuscript** |
| --- | --- | --- |
|  | Overall, the manuscript is well written and interesting. I applaud the authors for collecting such a diverse and large data set and providing interesting and well-done analyses. However, my reading indicated some points that should be addressed for publication, and I expect that some points might affect the findings (foremost the estimation method of factor analyses).  I hope that these comments contribute in publishing the manuscript. I am looking forward to read the updated version of the manuscript. | We appreciate the time and effort you dedicated to reviewing our manuscript and providing constructive feedback to help strengthen the final product. We respond below to each of your points. |
| **1** | Authors use ML estimation for the factor analyses, but the measure uses a 4-point rating scale. However, there is a discrepancy between the assumptions of ML (normality and continuous data distribution) that are violated by such response scales. This leads to biases and misestimations. Accordingly, the WLSMV estimator has been introduced to deal with such issues (see Brauer et al., 2023; for a discussion). The minimum requirement is that the data are re-analyzed with the WLSMV estimator and findings are provided to examine the degree of change that is caused by the estimator.  Brauer, K., Ranger, J., & Ziegler, M. (2023). Confirmatory factor analyses in Psychological Test Adaptation and Development: A non-technical discussion of the WLSMV estimator. Psychological Test Adaptation and Development, 4, 4-12. [https://doi.org/10.1027/2698-1866/a000034](https://aus01.safelinks.protection.outlook.com/?url=https%3A%2F%2Fdoi.org%2F10.1027%2F2698-1866%2Fa000034&data=05%7C02%7Cd.gucciardi%40curtin.edu.au%7C79b20d9f9cfe465ae49208dd2fae6cad%7C5a740cd757684d09ae13f706b09fa22c%7C0%7C0%7C638719148375144971%7CUnknown%7CTWFpbGZsb3d8eyJFbXB0eU1hcGkiOnRydWUsIlYiOiIwLjAuMDAwMCIsIlAiOiJXaW4zMiIsIkFOIjoiTWFpbCIsIldUIjoyfQ%3D%3D%7C0%7C%7C%7C&sdata=ffUJJthbBBHy9nSkE95cFin03Fiyke29Hc7sEVKgHgI%3D&reserved=0) | Thanks for raising this point. As noted in our manuscript, the 20 APP items are rated using a 5-point scale rather than a 4-point scale as you note in your feedback (lines 189-193).  *Clinical supervisors score each item, with reference to accompanying performance indicators, using a 5-point Likert scale (0 = infrequently/rarely demonstrates performance criteria to 4 = demonstrates most performance criteria to an excellent standard) to obtain a total score (out of 80), which is then represented as a percentage*.  We erroneously excluded key text from our description of the analytical model to indicate that we used ML estimation with robust standard errors, which we’ve updated in the revised submission as noted below.  *Our analyses relied on full information maximum likelihood estimation with robust standard errors (MLR) to utilise all data to estimate models, with listwise deletion for analyses where missing data existed on personal or contextual factors*.  We’re aware of the WLSMV estimator for categorical variables, which we considered originally but decided to proceed with MLR estimation because it provides access to Akaike Information Criteria (AIC), Bayesian Information Criteria (BIC), and sample sized adjusted BIC (ABIC) that can be used for model selection purposes. We also have reservations regarding the extent to which simulation evidence comparing MLR and WLSMV estimation – which is referenced in the Brauer et al. (2023) editorial – generalises to our context. Regarding Li’s (2016) simulations, for example:   1. The population model included 1 size for factor loadings (0.7), 1 size for the error term (0.51), and 1 size for the latent factor correlation (0.3). Essentially, the simulations are constrained to one simple representation of numerous possibilities. We have a much more complex model than the representation captured in this population model. 2. The sample sizes tested maxed out at 1000. We have a substantially larger sample size, which *generally but not always* comes with enhanced precision for statistical estimates. 3. The simulations utilised a confirmatory factor analysis framework only. We prioritised the penalised structural equation model, which makes fewer strict assumptions of the data (e.g., exactly zero cross-loadings) that are most aligned with ‘real-world’ data.   Nevertheless, we appreciate some readers might wish to know how our MLR results compare with those estimates obtained with WLSMV, so we have added these findings to the revised submission. We’ve provided these results in the supplementary material file S3 including model-data fit estimates and factor loadings. We’ve also included a footnote in the revised submission to alter readers to this additional information and context.  *Model-data fit and factor loading estimates obtained with WLSMV estimation are provided in supplementary material file 3. Overall, all models evidenced acceptable model-data fit according to CFI, TLI, and RMSEA values, except for the PSEM bifactor (4 items for professional) representation which did not converge as an unidentified model. Regarding our preferred PSEM 2-factor (4 items for professional and 16 items for clinical) solution, differences in factor loadings were minimal (0 – 0.15) and consistent with interpretations of intended factor loadings and cross-loading benchmarks between MLR and WLSMV estimation*.  Li, C. H. (2016). Confirmatory factor analysis with ordinal data: Comparing robust maximum likelihood and diagonally weighted least squares. *Behaviour Research Methods, 48*(3), 936–949. 10.3758/s13428-015-0619-7 |
| **2** | The evaluation of loadings is very lenient. I recommend to use a clear cut-off criterion to evaluate when a loading is substantial. The literature frequently refers .32 as a meaning loading. Adopting this approach would eliminate Item 4 (maybe also Item 2). However, I think a re-evaluation of the loadings should be done on basis of the results when using the WLSMV estimator. | We’re unsure why you raised this point, given the information we presented in the manuscript. First, we explicitly acknowledged effect size magnitudes for intended factor loadings and cross-loadings on non-intended factors in our statistical analysis section that contributed to our model selection process.  *Regarding model selection, we prioritised model superiority for lower values for the Akaike Information Criteria (AIC), Bayesian Information Criteria (BIC), and sample sized adjusted BIC (ABIC) [37]; and sound intended factor loadings (~ >.40) and small cross-loadings (~ <.20)*.  Second, in this statement, we acknowledged intended factor loadings of ~ >.40 as the priority for model selection, which is higher than your recommended .32 magnitude. If we adopted your proposed magnitude of .32 would mean that item 4 is retained unequivocally as an intended factor item for the professional domain (0.375) rather than excluded or considered borderline (see Table 3). Regarding item 2, perhaps you refer to the cross-loading on the clinical dimension (0.315) rather than intended factor loading on the professional dimension (0.468). |
| **3** | I have missed information about the reliability (McDonald's omega and retest-correlations over time). Since the data are available, these important findings should be provided. | Apologies, we erroneously excluded information on internal reliability of the latent factors in our previous submission; we’ve included this information in the revised submission in the statistical analysis and results sections, as noted below:  *We calculated omega (ω) as an estimate of internal reliability for latent factors of our preferred structural representation*.  *The latent factor correlation between the professional (ω = .80) and clinical (ω = .96) dimensions was moderately strong*.  Regarding test-retest reliability, we’ve decided against adding this information for several reasons. First, the exact time interval between assessments varies both within and between students, which likely adds ‘noise’ to any estimate. Second, the time interval between assessments generally exceeded several weeks for students, where we would expect some degree of observed change reflects true change in clinical performance. Third, our measurement invariance analyses provide insight into the most important consideration in contexts where we’d expect the target concept to change over time, namely the degree to which the same concept is captured equally over time and across contexts. |
| **4** | I have not found the items in verbatim. Please report them to allow others to use the instrument. | As noted in the methods section, the full 20-item APP is available in supporting information S2 (lines 186-189).  *The 20-item instrument covers seven key domains of physiotherapy practice, namely professional behaviour, communication, assessment, analysis and planning, intervention, evidence-based practice, and risk management (see Supporting Information S2) [3-5]*. |
