# Supplementary material for: The assessment of physiotherapy practice is a robust measure of entry-level physiotherapy standards: Reliability and validity evidence from a large, representative sample
Source: PLoS One. 2025 Apr 18;20(4):e0321397. doi: 10.1371/journal.pone.0321397 (PMC12007712; doi:10.1371/journal.pone.0321397)
Supplement: Attachment — Submitted filename: PONE-D-24-38498R2_response to reviewers.docx [file pone.0321397.s007.docx]

**REVIEWER 2**

|  | **Reviewer’s Comments** | **Our Response and Location in Revised Manuscript** |
| --- | --- | --- |
| **1** | In response to my comment #2, which raises concerns about the interpretation of the loadings, you ask why I raised this point. First, using the widely used cut-off of .32 allows to identify cross-loadings several cross-loadings in your scale that are not detected with a comparatively lenient cut-off of .40. Secondly, you do not present or discuss consequences that follow from the loadings not meeting cut-offs or showing substantial cross-loadings. The standard would be to exclude items that do either not load on the respective factor or show cross-loadings and compute a new factor model, which is then evaluated regarding fit and loadings. This is done until a final well-defined model is available. The discussion should then highlight which items were excluded and why, and what the final version of the measurement instrument is. | Apologies, we misinterpreted your comment on the previous version, where you wrote:  “*The evaluation of loadings is very lenient. I recommend to use a clear cut-off criterion to evaluate when a loading is substantial. The literature frequently refers .32 as a meaning loading. Adopting this approach would eliminate Item 4 (maybe also Item 2). However, I think a re-evaluation of the loadings should be done on basis of the results when using the WLSMV estimator*.”  We interpreted this statement as being applicable only to intended factor loadings rather than cross-loadings. Regarding the widely used cut-off of .32, we traced this reference value to Costello and Osborne (2005), who cited Tabachnick and Fidell (2001), where they stated:  “*Tabachnick and Fidell (2001) cite .32 as a good rule of thumb for the minimum loading of an item, which equates to approximately 10% overlapping variance with the other items in that factor*.”  As noted in our previous response, we explicitly acknowledged effect size magnitudes for intended factor loadings and cross-loadings on non-intended factors in our statistical analysis section that contributed to our model selection process.  *Regarding model selection, we prioritised model superiority for lower values for the Akaike Information Criteria (AIC), Bayesian Information Criteria (BIC), and sample sized adjusted BIC (ABIC) [37]; and sound intended factor loadings (~ >.40) and small cross-loadings (~ <.20)*.  Regarding your second point about the discussion of cross-loadings, we dedicated an entire paragraph to this exact point:  “*Despite the firm conclusion regarding the dimensionality of entry-level physiotherapy performance, there are important nuances to the data which require consideration for future use. On the surface, the PSEM 2-factor (four items for professional) model provided the best representation of entry-level competence, yet some item level data were less optimal. Specifically, item 4 “demonstrates collaborative practice” had almost identical factor loadings across professional and clinical dimensions (0.375 vs 0.415 respectively), whereas three items (2 – commitment to learning, 5 – verbal and non-verbal communication, and 20 – identifies adverse events/near misses and minimises risk associated with assessment and interventions) loaded higher on their intended factor relative to the unintended factor, but failed to meet one or both criteria (>.40 on intended and <.20 on unintended factor). Performance indicators within item 4 include “works collaboratively & respectfully with support staff” and “collaborates with the health care team & client to achieve optimal outcomes”. At face value, these likely cut across most, if not all aspects, of entry-level physiotherapy performance. Commitment to learning (item 2) includes some performance indicators that are subjective in nature, such as “takes responsibility for learning…” and “demonstrates self-evaluation…”. Items that require less subjectivity exhibited higher factor loadings, emphasising the need for clarity in APP item indicators, especially considering the dynamic nature of contemporary healthcare settings [20]. Given the 2023 update in physiotherapy practice standards within ANZ, an examination of item content may enhance conceptual clarity and scoring precision [17].*”  Overall, your feedback implies that we should rely solely on statistical criteria to decide which items should be retained to reflect the content space of the latent factors. As implied in the text above, our position is that statistical criteria should be considered alongside theoretical or conceptual issues for making inferences regarding the suitability of item content. For example, we’d expect few subject matter experts would disagree that collaborative practice is central to the representation of entry-level physiotherapy performance. Excluding this item from the final pool is therefore likely to diminish content validity. Rather, we propose that our data should provide motivation for future work on the refinement of such items to minimise ambiguity and maximise conceptual precision.  How exactly have we addressed your feedback in this revised submission?   First, we’ve acknowledged the proposal to rely on a factor loading of ≥.32 for meaningful loadings as being prevalent in the literature, and elaborated on our rationale to rely on an inference framework characterised by sound intended factor loadings (~ >.40) and small cross-loadings (~ <.20).  *One reviewer recommended we use the rule of thumb of ≥.32 for meaningful factor loadings [39]. However, we preferred an approach where the meaningful intended factor loadings can be differentiated quantitatively from meaningful cross-loadings. Said differently, we prioritised an inference framework where the difference in magnitude of loadings for each item on their intended versus unintended factor were approximately .20*.  Second, we’ve expanded on the discussion point regarding the salience of cross-loadings for the APP. Specifically, we make it explicit that our position regarding the retainment of items should balance statistical criteria alongside conceptual or theoretical considerations.  *Despite the firm conclusion regarding the dimensionality of entry-level physiotherapy performance, there are important nuances to the data which require consideration for future use. On the surface, the PSEM 2-factor (four items for professional) model provided the best representation of entry-level competence, yet some item level data were less optimal. Specifically, item 4 “demonstrates collaborative practice” had almost identical factor loadings across professional and clinical dimensions (0.375 vs 0.415 respectively), whereas three items (2 – commitment to learning, 5 – verbal and non-verbal communication, and 20 – identifies adverse events/near misses and minimises risk associated with assessment and interventions) loaded higher on their intended factor relative to the unintended factor, but failed to meet one or both criteria (>.40 on intended and <.20 on unintended factor). Performance indicators within item 4 include “works collaboratively & respectfully with support staff” and “collaborates with the health care team & client to achieve optimal outcomes”. At face value, these features likely cut across most, if not all aspects, of entry-level physiotherapy performance. Commitment to learning (item 2) includes some performance indicators that are subjective in nature, such as “takes responsibility for learning…” and “demonstrates self-evaluation…”. Items that require less subjectivity exhibited higher factor loadings, emphasising the need for clarity in APP item indicators, especially considering the dynamic nature of contemporary healthcare settings [20]. Relying solely on statistical criteria to make inferences regarding item selection and retainment would likely mean that we’d need to compromise the content validity of entry-level physiotherapy performance in some way. Rather than throw the baby out with the bathwater, future work is required to refine these ambiguous items to maximise their conceptual clarity and scoring precision. The conceptual feature of ‘demonstrates collaborative practice’, for example, could be partitioned into separate items that specify with precision exactly what this collaborative approach looks like for professional and clinical elements of performance. Item enhancements could also align with item content with the 2023 update in physiotherapy practice standards within ANZ [17]*.  Costello AB, Osborne J. Best practices in exploratory factor analysis: four recommendations for getting the most from your analysis. Pract Assess Res Evaluation. 2005;10(1). doi: <https://doi.org/10.7275/jyj1-4868>  Tabachnick, B. G., & Fidell, L. S. (2001). *Using Multivariate Statistics*. Boston: Allyn and Bacon. |
