# Supplementary material for: The assessment of physiotherapy practice is a robust measure of entry-level physiotherapy standards: Reliability and validity evidence from a large, representative sample
Source: PLoS One. 2025 Apr 18;20(4):e0321397. doi: 10.1371/journal.pone.0321397 (PMC12007712; doi:10.1371/journal.pone.0321397)
Supplement: Attachment — Submitted filename: PONE-D-24-38498.R4_response to reviewers.docx [file pone.0321397.s008.docx]

**REVIEWER 4**

|  | **Reviewer’s Comments** | **Our Response and Location in Revised Manuscript** |
| --- | --- | --- |
| **1** | You have presented a well-structured and detailed response that shows that you thoroughly considered the feedback from of Reviewer #2. You have clarified your rationale for the cut-offs you selected for factor loading, described how you dealt with cross-loadings and included a plan for refining ambiguous items as a future research area. I support your approach of balancing between statistical rigor and consideration for conceptual validity.  However, to make your argument stronger, you may discuss whether you considered Reviewer #2’s suggestion to recompute the factor model iteratively until stability was reached, how that looked like and why you eventually stuck to the current approach. In addition to this, you could provide a clearer framework for deciding to keep items with cross loadings. This could be for example through specifying a threshold or guideline that weighs conceptual importance over statistical considerations. This would be very helpful for other researchers seeking to replicate your approach.  I recommend a minor revision for these refinements, which will enhance clarity and transparency without requiring substantial changes, given the strength of the current version of your manuscript. | Thanks for your review. We appreciate the time you’ve dedicated to reviewing our work, including the iterative changes and responses from previous reviews, as well as your recommendation to strengthen the final manuscript.  For context, here is the comment from Reviewer 2:  “*In response to my comment #2, which raises concerns about the interpretation of the loadings, you ask why I raised this point. First, using the widely used cut-off of .32 allows to identify cross-loadings several cross-loadings in your scale that are not detected with a comparatively lenient cut-off of .40. Secondly, you do not present or discuss consequences that follow from the loadings not meeting cut-offs or showing substantial cross-loadings. The standard would be to exclude items that do either not load on the respective factor or show cross-loadings and compute a new factor model, which is then evaluated regarding fit and loadings. This is done until a final well-defined model is available. The discussion should then highlight which items were excluded and why, and what the final version of the measurement instrument is*.”  Using the same sample to test an initial structure and revise that structure iteratively to land on an acceptable solution would be considered a questionable research practice (Crede & Harms, 2019). We’ve included this footnote in the revised submission to articulate our position: *One reviewer suggested we remove these poorly fitting items according to statistical criteria alone then recalculate the factor model, and do so iteratively until a well-defined model is achieved. In essence, iteratively modifying the model based on observed loadings within the same dataset can capitalise on chance variation, leading to overfitting and compromised generalisability. This approach resembles post hoc model specification, where decisions about item retention and factor structure are made after examining the data rather than being guided by a priori theoretical or empirical criteria. The iterative removal of poorly fitting items based on statistical criteria alone, followed by re-estimation of the model with the same sample, can artificially inflate model fit and lead to a final structure that may poorly generalise to other samples. Most importantly, ignoring conceptual considerations for the removal of items would inevitably weaken our confidence in the content validity of the Assessment of Physiotherapy Practice (APP). Removing items with uncertain statistical properties in our analyses – “demonstrates collaborative practice”, “commitment to learning”, “verbal and non-verbal communication”, and “identifies adverse events/near misses and minimises risk associated with assessment and interventions” – would effectively remove essential content that is required for entry-level physiotherapy performance*.  Regarding your recommendation to strengthen our justification of the item retention criteria, we’ve updated the explanation originally provided in footnote 2 and repositioned this information to the main body of text (lines 237-246):  *In so doing, we preferred an approach where meaningful intended factor loadings can be differentiated quantitatively from meaningful cross-loadings, rather than rely on the rule of thumb of ≥.32 for meaningful factor loadings [39]. Said differently, we prioritised an inference framework where the difference in magnitude of loadings for each item on their intended versus unintended factor were approximately .20. Although we monitored model-data fit indices to evaluate the overall model quality, we focused primarily on conceptually informed item retention because purely statistical benchmarks can undermine construct representation (e.g., breadth, depth). Thus, when an item with a statistically borderline loading represented a theoretically essential aspect of the target construct, we prioritised content validity over strict adherence to cutoff values*.  Finally, we’ve explicitly acknowledged in the discussion section that ongoing psychometric evaluations of the APP in its current form and/or any refinements to the content space are required to sustain our confidence in this operationalisation of entry-level physiotherapy performance.  *As reliability and validity are properties of test scores rather than instruments themselves, we advocate for ongoing validation work on the current version of the APP or any item refinements to the conceptual space*.  Crede, M., & Harms, P. (2019). Questionable research practices when using confirmatory factor analysis. *Journal of Managerial Psychology*, *34*(1), 18-30. doi: 10.1108/jmp-06-2018-0272 |
